# Phase I clinical trials for cardiovascular diseases in Europe: analysis of trends, distribution, and regulatory context

**DOI:** 10.3389/fphar.2026.1774988

**Published:** 2026-05-12

**Authors:** Stefano Grosdani, Arianna Bertolani, Giorgio Reggiardo, Nicola Cosentino, Giancarlo Marenzi, Piergiuseppe Agostoni, Giulio Pompilio, Arianna Pani, Marco Scatigna

**Affiliations:** 1 Centro Cardiologico Monzino, IRCCS, Milan, Italy; 2 Postgraduate School of Clinical Pharmacology and Toxicology - Department of Medical Biotechnology and Translational Medicine, University of Milan, Milan, Italy; 3 Empowering Life Science Education, Evidence and Engagement (ELSE) S.r.l., Milan, Italy; 4 Consorzio per Valutazioni Biologiche e Farmacologiche, Pavia, Italy; 5 Department of Oncology and Hematology-Oncology, University of Milan, Milan, Italy

**Keywords:** cardiometabolic disorders, cardiovascular diseases, drug development, phase I clinical trials, regulatory framework

## Abstract

**Introduction:**

Cardiovascular diseases and their major metabolic risk factors remain the leading contributors to morbidity and mortality in Europe. Despite the growing burden of cardiometabolic conditions and the critical role of early-phase research in drug development, the landscape of Phase I clinical trials in this therapeutic area has not been systematically explored in Europe.

**Methods:**

We analysed Phase I clinical trials registered on ClinicalTrials.gov from inception to December 2024, focusing on five European countries (France, Germany, Italy, Spain, and the United Kingdom). After applying disease-specific filters and excluding duplicates, device and dietary supplement studies, 488 trials were retrieved. Conditions were classified into 12 ICD-11-based disease groups and into two macro-categories: cardiovascular diseases (CVDs) and cardiovascular risk factors (CVRFs).

**Results:**

Of the 488 trials, 35% targeted CVDs and 65% CVRFs. Diabetes mellitus was the most frequently studied condition (48%), followed by obesity (9%) and heart failure (7%). Most trials (84%) were industry-sponsored, with seven companies accounting for one-third of studies. Germany and the United Kingdom conducted the most trials (262 and 170 trials), while Italy the fewest (20). Southern countries focused predominantly on CVDs, whereas northern countries more on CVRFs. Trials involving ATMPs and rare diseases were proportionally more common in Spain and Italy. Overall, 51% of studies enrolled healthy volunteers, though none in Italy.

**Conclusion:**

Significant geographic disparities and a declining trend in early-phase cardiometabolic research may weaken Europe’s competitiveness in drug development. Strategic investments in infrastructure, regulatory harmonization, and public-private collaboration are needed to reverse the recent trend.

## Highlights

- The study provides first analysis of Phase I trials for CVDs in Europe.
- Marked disparities in trial volume, characteristics and sponsorship emerged.
- Overall growth until 2015, followed by a significant decline in phase I activity.
- Europe needs targeted policies to strengthen early-phase competitiveness.

## Introduction

Cardiovascular diseases (CVDs) remain the leading cause of mortality in Europe, accounting to more than 60 million potential years of life lost annually (Townsend et al., 2022). The well-established interplay between CVDs and metabolic disorders has prompted a paradigm shift toward recognizing cardiometabolic diseases as a unified group of interrelated conditions. Metabolic pathologies such as hypertension, dyslipidaemia, obesity and diabetes represent the major modifiable risk factors for cardiovascular morbidity and mortality (CDC, 2024).

The situation is aggravated by the observation of a globally increasing prevalence of these metabolic conditions, driven largely by sedentary lifestyles, unhealthy diets, and aging populations (Chew et al., 2023). In the European Region, 1 in 10 people are estimated to have diabetes, over 90% of which are type 2 diabetes (T2D), of which 1 in 3 is undiagnosed (Europe, 2019). Meanwhile, hypertension, obesity and elevated total cholesterol affect, respectively, around 22%, 22.5% and more than 50% of the EU population (Timmis et al., 2022), reinforcing the urgent need for novel and effective treatment strategies.

The development of novel pharmacological treatments to address these diseases relies on the clinical trial process, with phase I clinical trials representing a fundamental step in the development of new drugs (Muglia and DiGiovanna, 1998). Indeed, phase I clinical trials represent the initial interface between an investigational drug and human subjects, typically involving a small cohort, up to a few dozens, of healthy volunteers or patients. Their primary objectives are to assess the safety and tolerability of the investigational product, while investigating pharmacokinetics parameters. Participants receive very low starting doses of the novel drug, which are usually gradually increased in order to find the recommended phase II dose, through intensive monitoring of participants to detect any adverse effects.

Due to the inherent risks associated with administering a novel agent to humans for the first time, these studies are governed by a stringent regulatory framework designed to ensure participants’ safety.

At the European Union level, the main regulatory references include: (i) EMA Guideline on Strategies to Identify and Mitigate Risks for First-in-Human and Early Clinical Trials with Investigational Medicinal Products (2017) that serves as guidance to the transition from preclinical to clinical phases by identifying potential risk factors and providing instructions on dose selection, escalation, and study design (EMA, 2017); (ii) EMA Annex V–to guidance for the conduct of good clinical practice inspections–Phase I units (2022) that provides guidance for the preparation of Good Clinical Practice (GCP) inspections of phase I units, focusing on critical aspects such as the protocol, ethical and regulatory approval, recruitment and informed consent, adverse event management, and confidentiality (Annex V, 2022).

For reference, the U.S. Food and Drug Administration (FDA) has issued detailed guidelines on first-in-human and early-phase clinical trials, covering topics ranging from good manufacturing practices for investigational new drugs (INDs) (DE, 2023), to the information required for IND Phase I applications and dose selection (INDS, 2023; FDA, 2000). In parallel, China has introduced regulatory reforms since 2015 to accelerate early-phase drug development (Song et al., 2019). Of note, the current European regulatory framework governing early-phase trials was significantly updated in response to historical adverse events, in particular to the tragic death of a volunteer in January 2016 following the administration of a novel fatty acid amide hydrolase inhibitor (van Gerven et al., 2018).

Previous analyses have explored trends in early-phase clinical trials across therapeutic areas (Di Tonno et al., 2022). However, despite the imperative need for new therapeutics for CVDs, no study, to our knowledge, has ever investigated the state of phase 1 clinical trials in Europe for the treatment of CVDs and related metabolic risk factors.

The objective of the present study was, therefore, to provide a descriptive and quantitative analysis of phase 1 clinical trials conducted in Europe for the treatment of CVDs and their major risk factors, focusing on five selected European countries. Using ClinicalTrials.gov as the data source, we examined temporal trends, geographic distribution, and key trial characteristics to better characterize the European landscape of early-phase cardiometabolic research and to provide valuable insights for stakeholders involved in drug development and regulatory policy.

## Methods

The methodology adopted for this analysis follows the model published by Di Tonno et al. (Di Tonno et al., 2022). The ClinicalTrials.gov database was reviewed and a CSV dataset (updated from inception to December 2024) comprising 3,107 registered phase 1 clinical studies on ClinicalTrials.gov of five selected European countries (France, Germany, Italy, Spain and the UK) was downloaded. The countries were selected based on similarities in terms of demographic and Gross Domestic Product data available on official government websites. The selection criteria applied included the following diseases/therapeutic areas: cardiology, heart diseases, diabetes, obesity, endocrinology, metabolic disorders, hypercholesterolemia, hypertension, hyperglycemia, hypertriglyceridemia, and dyslipidemia. Additional filters were applied to include only studies in the early phase or phase I stage and to distinguish between those involving healthy volunteers and patients. No restrictions were set regarding the time frame of trial execution. The full search strategy is provided in the Supplementary Material.

The study selection process is shown in Supplementary Figure S1. We first manually reviewed the downloaded dataset to obtain homogeneous data and perform the analysis. After removing duplicate studies and those conducted on medical devices, procedures or dietary supplements from the downloaded dataset, we obtained a final cleaned dataset of 488 registered phase 1 clinical trials. For each trial, the investigated condition was assessed, with specific attention to the involvement of ATMPs and rare diseases (RDs). Furthermore, the medical conditions explored across the studies were classified into 12 disease groups according to the ICD-11 (International Classification of Diseases 11th Revision) taxonomy and a short abbreviation code was applied to facilitate data management and statistical analyses. These groups were subsequently consolidated into two overarching macro-categories: CVD and cardiovascular risk factors (CVRFs) (Supplementary Table S1).

For the overall European analysis, Phase I clinical trials conducted across multiple countries of interest were excluded to prevent data duplication. Conversely, in the country-specific analyses, all trials involving multiple countries were considered to preserve national-level specificity.

As regards the funding source, the total number of phase 1 clinical trials funding was divided into “Industry” and “Other” per country. The latter, based on the ClinicalTrials.gov database filters, comprises individuals, universities, organizations, NIH and other U.S. federal organizations.

### Statistical analysis

Univariate linear regression analysis was used to evaluate longitudinal trends in the total number of studies in the period 1995–2024. The slope of the regression line indicates the average increase or decrease in the number of studies per unit of time (year). Using this approach, it is possible to evaluate longitudinal trends across different periods (1995–2015 and 2015–2024) to determine if there was a statistically significant increase or decrease in the number of studies per year. All statistical tests were two-sided and performed using a significance (alpha) level of 0.05. Data were analysed using the Statistical Package for the Social Sciences (SPSS) software (IBM SPSS Statistics for Windows, version 29.0, IBM Corporation, Armonk, NY, United States).

## Results

**FIGURE 1:**
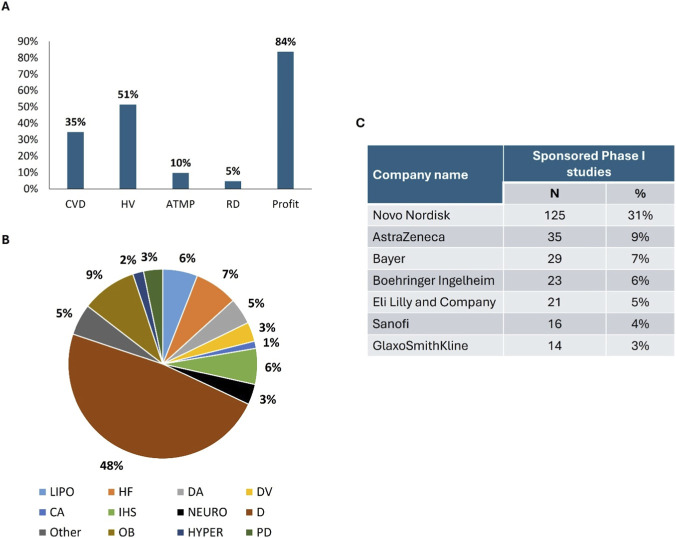
Primary characteristics, most frequently investigated conditions, and percentage of industry-sponsored trials in the analyzed phase I studies. Phase I activity is concentrated in industry-sponsored trials mainly focused on diabetes and other cardiovascular risk factors, with limited representation of ATMP and rare disease studies. Panel **(A)** Main features of the evaluated trial Panel **(B)** Distribution of disease categories Panel **(C)** Sponsorship concentration Abbreviations: CVD = cardiovascular disease; HV = healthy volunteer; ATMP = advanced therapeutic medicinal product; RD = rare diseases; LIPO = Disorders of lipoprotein metabolism or certain specified lipidaemias; OB = Nutritional disorders–Overweight, obesity or specific nutrient excesses; D = Endocrine diseases–Diabetes mellitus; HYPER = Hypertensive diseases; HF = Heart failure; DA = Diseases of arteries or arterioles; DV = Diseases of veins; CA = Cardiac arrhythmias; IHS = Ischaemic heart diseases; PD = Pulmonary heart disease or diseases of pulmonary circulation; NEURO = Cerebrovascular diseases.

From the inception of ClinicalTrials.gov through December 2024, a total of 488 Phase I clinical trials were registered in the areas of cardiovascular diseases (169; 35%), and cardiovascular risk factors (319; 65%). Among the 12 disease groups of focus, the most frequently investigated conditions were diabetes (48%), followed by obesity (9%), heart failure (7%), and hyperlipoproteinaemia and ischaemic heart diseases (6%) (Figures 1A,B).

Of the total 488 studies, 408 (84%) were sponsored by 92 pharmaceutical companies. Seven of these companies each supported at least 10 studies. Novo Nordisk emerged as the leading sponsor, accounting for 125 trials (31%), followed by AstraZeneca (9%), Bayer (7%), Boehringer Ingelheim (6%), Eli Lilly (5%), Sanofi (4%), and GlaxoSmithKline (3%) (Figure 1C).

Analysis of the full study sample revealed that 10% involved ATMPs and 5% targeted rare diseases. These trials were predominantly patient-based, with 100% of ATMP studies and 78% of RD studies enrolling patient populations. Notably, 251 trials (51%) out of 488 were conducted in healthy volunteers.

In the absence of time filters during data extraction, analysis of the final database showed that the earliest study dated back to 1995 and was conducted in the United Kingdom. In the country-specific analyses, all retrieved Phase I clinical trials, including those involving multiple countries, were considered to ensure national-level specificity. This resulted in a total of 540 trials related to cardiovascular diseases and its risk factors.

**FIGURE 2:**
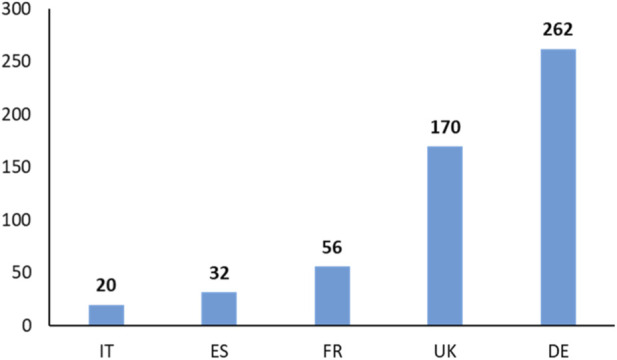
Total number of phase I trials by country. Phase I cardiometabolic trial activity is highly concentrated in Germany and the United Kingdom, with markedly lower activity in Italy and Spain. Abbreviations: IT = Italy; ES = Spain; FR = France; UK = United Kingdom; DE = Germany.

**FIGURE 3:**
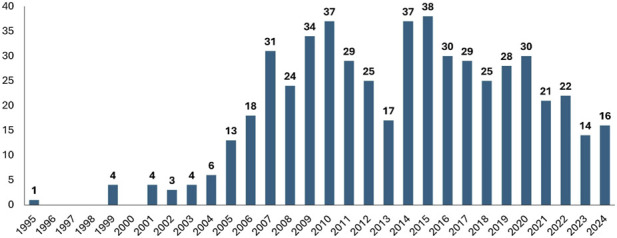
Temporal trend in phase 1 trials execution in the selected European countries. Phase I trial activity increased steadily until 2015, followed by a significant decline across the selected countries.

Germany emerged as the leading country in terms of trial volume, with 262 studies, followed by the United Kingdom with 170. In contrast, Italy recorded the lowest number, with only 20 studies conducted during the period considered (Figure 2). Two different temporal trends in the conduct of phase I trials are evident (Figure 3). When evaluating the entire period from 1995 to 2024 (Supplementary Figure S2), a significant positive linear trend is evident (p < 0.001), with a correlation between the number of studies and time (R^2^ = 0.469) and an estimated annual increase of 1.01 Phase I trials (Supplementary Table S2). However, this overall growth is driven by the period from 1995 to 2015, during which a strong and significant positive trend is seen (p < 0.001; R^2^ = 0.792), corresponding to an average annual increase of 2.08 trials (Supplementary Table S3). In contrast, from 2015 onward, the trajectory reverses. A significant negative trend emerges (p < 0.001; R^2^ = 0.789), with an estimated annual decline of 2.15 trials (Supplementary Table S4).

The distribution of Phase I clinical trials across the two defined macro-categories (cardiovascular diseases and related risk factors) and the 12 disease groups (hyperlipoproteinaemia, overweight/obesity, hypertension, diabetes mellitus, heart failure, diseases of arteries or arterioles, diseases of veins, cardiac arrhythmia, ischaemic heart diseases, cerebrovascular diseases, pulmonary heart disease or diseases of pulmonary circulation and other) varied notably among countries (Supplementary Figure S4).

**FIGURE 4:**
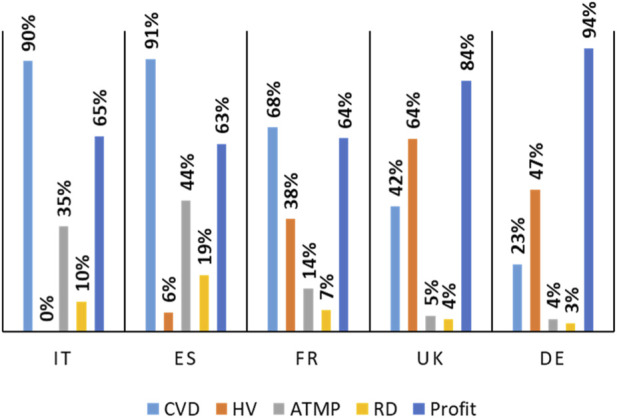
Primary phase I trials characteristics by country. Substantial inter-country differences exist in trial type, sponsorship, ATMP involvement, and use of healthy volunteers. Abbreviations: CVD = cardiovascular disease; HV = healthy volunteer; ATMP = advanced therapeutic medicinal product; RD = rare diseases; IT = Italy; ES = Spain; FR = France; UK = United Kingdom; DE = Germany.

As shown in Figure 4, in Italy and Spain, the vast majority of studies was focused on CVDs, accounting for 90% and 91% of all trials, respectively, whereas only a small proportion investigated the CVRFs (10% and 9%). France showed a more balanced distribution, with 32% of trials addressing CVDs and 68% related to CVRFs. In the United Kingdom, the proportion of CVD-related phase 1 trials was higher (58%) than those on CVRFs (42%). Germany had the largest number of CVD studies (77% of trials), while only 23% focused on CVRFs. Overall, these findings indicate that early-phase cardiovascular research activity is highly heterogeneous across European countries, with southern regions (Italy and Spain) more focused on metabolic and vascular risk factors, and northern regions (UK and Germany) showing greater involvement in trials directly targeting cardiovascular diseases.

Disease specific individual-country-level data is shown in Supplementary Figure S4. In Italy, most trials were classified under diseases of arteries or arterioles (DA) and diseases of veins (DV) (20% each), followed by heart failure (HF, 15%), while studies addressing cardiovascular risk factors were less frequent (hyperlipoproteinaemia (LIPO) and diabetes mellitus (D), 5%) or absent (overweight/obesity (OB) and hypertension (HYPER)).

In Spain, a similar pattern was observed, with an equal contribution (16% each) of trials in DA, DV, ischaemic heart diseases (IHS), and cerebrovascular diseases (NEURO), whereas no trials were registered for LIPO or HYPER, and only a few (3%) for OB and cardiac arrhythmias (CA).

In France, trials were more evenly distributed across disease groups, with the largest share of studies (16%) on D and NEURO, followed by DV, 13%, whereas no trials were registered for CA.

In the United Kingdom, trials most frequently targeted diabetes mellitus (D, 34%), overweight/obesity (OB, 11%), and hyperlipoproteinaemia (LIPO, 11%), while other groups such as hypertension (HYPER), diseases of arteries or arterioles (DA), diseases of veins (DV), and cardiac arrhythmias (CA) were less represented (2%).

Finally, in Germany, the overwhelming majority of trials focused on diabetes mellitus (D, 65%), with the lowest proportions (1%) in hypertension (HYPER) and cerebrovascular diseases (NEURO).

Overall, diabetes mellitus emerged as the most represented disease group across all countries (44% of total Phase I trials), whereas studies in pulmonary heart disease, hypertension, and cardiac arrhythmias remained relatively scarce (≤5%). This heterogeneity suggests that national research priorities and sponsor interests differ markedly across Europe, with southern countries focusing more on vascular disorders and northern countries displaying greater activity in metabolic and endocrine-related Phase I studies.

Main trial features divided by country are highlighted in Figure 4. Germany and the United Kingdom also led in terms of industry-sponsored (profit) studies, with 247 and 142 trials, respectively. These numbers correspond to the highest proportions of profit-sponsored trials relative to national totals, 94% in Germany and 84% in the UK. Conversely, Italy demonstrated the weakest capacity to attract industry sponsorship, with only 13 sponsored trials, representing 65% of the total conducted nationally.

Regarding innovation-driven research, Spain and Italy reported the highest shares of trials involving ATMPs, representing 44% (Di Tonno et al., 2022) and 35% (EMA, 2017) of their respective national totals. In contrast, the United Kingdom (5%; 9) and Germany (4%; 11) reported considerably lower proportions. Spain also led in the proportion of studies focusing on rare diseases (19%; 6), whereas the UK and Germany had the lowest shares, 4% (EMA, 2017) and 3% (EMA, 2017), respectively, despite conducting the highest overall number of trials. Although Italy and Spain had lower overall trial volumes (Figure 2), they exhibited higher proportional involvement in ATMP and rare disease trials (Figure 4).

Lastly, the United Kingdom stood out for the highest proportion of studies conducted in healthy volunteers (64% of all national studies). In stark contrast, no studies involving healthy volunteers were identified in Italy within the considered therapeutic areas.

## Discussion

This study provides the first comprehensive analysis of Phase I clinical trials conducted in Europe for the treatment of cardiovascular diseases and their major risk factors. Across five selected European countries, we identified 488 trials focusing on these interrelated disease areas. Although this analysis does not directly measure regulatory timelines or administrative burden, it offers quantitative indicators of early-phase performance that may reflect underlying infrastructural and operational differences between countries.

Our analysis highlights two key observations: (Townsend et al., 2022): marked disparities exist between the selected European countries, which may reflect a broader gap between northern and southern Europe, and (CDC, 2024) a declining trend in Phase I clinical trials for CVDs and CVRFs has emerged.

Marked inter-country heterogeneity was observed in trial volume and characteristics. Germany and the United Kingdom conducted the majority of Phase I trials and exhibited the highest proportions of industry-sponsored studies. In contrast, Italy conducted the fewest trials overall and did not report any Phase I studies enrolling healthy volunteers within the investigated therapeutic areas. Taken together, these findings may indirectly signal differences in early-phase capacity. High trial volume, strong industry participation, and diversification of trial types may serve as proxies of a well-established early-phase ecosystem, whereas lower activity and diversification may indicate structural or procedural barriers.

Differences in national research investment may also contribute to this disparity. Publicly available data indicate that Germany and the United Kingdom consistently report higher absolute and proportional pharmaceutical R&D expenditures compared with several southern European countries (EFPIA, 2025). While our dataset does not directly measure R&D spending or facility density, the alignment between higher national research investment and greater Phase I activity observed in our analysis supports the interpretation that resource distribution plays a meaningful role.

Our longitudinal analysis demonstrated a statistically significant increase in Phase I cardiometabolic trials until 2015, followed by a marked decline thereafter. This trend is consistent with an overall decrease in early-phase studies across Europe (EFPIA, 2024), as well as with a more general decline in clinical research extending to phase II and III trials. This trend may reflect sponsors’ strategic decision to conduct early-phase studies in China and other parts of Asia, where regulatory and financial conditions are perceived as more favourable (Murphy, 2026).

These regions have implemented substantial regulatory reforms aimed at streamlining drug development. In China, changes introduced in 2015 with the “State Council Circular No. 44” (MFA, 2021) have notably reduced the timeline for Investigational New Drug application approvals. The median regulatory review time is now approximately 87 working days, and more than half of approved trials begin patient enrollment within 6 months (Zhang et al., 2024; Su et al., 2022). While this level of efficiency is now comparable to that of European countries, both regions continue to lag behind the USA in terms of trial initiation speed (Joppi et al., 2020).

Moreover, while USA and China have long benefited from a centralized approval process, avoiding the fragmentation that has historically hindered clinical trial initiation in Europe, only recently was the European Union able to address this issue through the introduction of the Clinical Trials Regulation (EU CTR) (EMA, 2015), which aims to streamline approvals. Notably, the UK is no longer included this process.

Financial considerations further reinforce the attractiveness of Asian regions. The cost of conducting a Phase I clinical trial in China is estimated at between 0.3 and 0.8 million USD, a fraction of the 3 to 5 million USD typically required in the United States or Europe (Nature, 2012). These cost disparities offer an attractive incentive for sponsors to relocate early-phase research activities to lower-cost countries while accessing a large population of treatment-naïve patients.

Consistent with this, our findings regarding sponsorship concentration–where seven companies accounted for approximately one-third of all identified trials–suggest that early-phase cardiometabolic research in Europe is dominated by large multinational sponsors. This pattern may reflect the challenges that smaller or emerging developers may experience due to high operational costs. In contrast, the Chinese clinical trial landscape is dominated by locally sponsored trials (Murphy, 2026), potentially reflecting lower development costs.

Of note, the observed declining trend is not driven by reduced clinical need or market interest. On the contrary, the cardiometabolic sector is expected to experience robust growth, with the global market projected to expand from $264 billion in 2022 to approximately $370 billion by 2027 (Gores and Lutzmayer, 2000). This growth aligns with the anticipated increase in prevalence of cardiometabolic conditions, particularly in Western countries, driven by increasingly sedentary lifestyles and calorie-dense diets. This contrast between a declining early-phase trials landscape in Europe versus a thriving global market suggests a progressive erosion of Europe’s competitiveness in early-phase development and underscores a potentially missed strategic opportunity. A strong cardiometabolic early-phase clinical activity could reverberate into more robust pipelines, innovation capacity and faster access to new therapeutics (EFPIA, 2024).

An important finding of this study is that countries with lower overall Phase I trial volume–particularly Italy and Spain–exhibited a disproportionately high involvement in ATMPs and rare disease studies. In absolute terms, the number of these trials was comparable to that observed in higher-volume countries, suggesting that innovation-driven research may develop independently of overall industry-sponsored trial activity. Given that rare diseases and ATMPs are increasingly recognized as priority areas for both academia and industry, this specialization may represent an opportunity for countries to strengthen their competitiveness in pharmaceutical research.

Moreover, enhancing collaboration between academia, industry, and regulatory bodies could also address another disparity that emerged from our analysis, namely, the uneven distribution of trials between disease areas. While diabetes and obesity represent today areas of high innovation, cardiovascular research faces stagnation. The complexity of cardiovascular diseases and the existence of well-established therapies lead to the need for large, long and costly trials with hard clinical endpoints, discouraging sponsors from early phase investment. Initiatives such as Horizon Europe are moving in this direction by funding collaborative research networks (Horizon Europe, 2026).

Taken together, the observed geographic concentration of trials (Figure 2), the post-2015 decline (Figure 3), and the predominance of large companies’ sponsorship (Figure 1A) highlight the need for targeted policy interventions to strengthen Europe’s early-phase research ecosystem. Countries with established high-volume environments may benefit primarily from regulatory optimization and operational efficiency, whereas countries with lower overall activity but demonstrated innovation potential may strategically invest in expanding early-phase infrastructure and specialized expertise. These approaches could enhance sponsor engagement and contribute to a more balanced and competitive European research landscape overall.

The observed findings align with concerns raised in European institutional and industry reports emphasizing the need to restore Europe’s competitiveness in pharmaceutical research. Recommended measures include modernizing intellectual property frameworks, streamlining and harmonizing clinical trial processes, reducing administrative burden, fostering adoption of innovative methodologies and technologies, and reinforcing patient-centric approaches (Accelerating Clinical Trials in the EU (ACT EU); EU; EFPIA, 2023). The implementation of the Clinical Trials Information System (CTIS) following the 2022 “Accelerating Clinical Trials in the EU (ACT EU)” (Horizon Europe, 2026) initiative marks a pivotal step toward a unified regulatory framework within the EU. Although the full impact of CTIS and ACT EU remains to be evaluated, preliminary data are encouraging. Member States that rapidly adopted the system, such as Spain, have demonstrated improved performance in both trial initiation and patient recruitment metrics (EFPIA, 2024).

Although this study focuses on CVDs and CVRFs, similar trends of declining early-phase activity extend to other therapeutic areas (EFPIA, 2024). Given the central role of Phase I trials in translational medicine and R&D productivity, sustained erosion of early-phase capacity may have implications for innovation output, therapeutic diversity, and timely patient access to new treatments. Ensuring a competitive, innovation-friendly early-phase environment is therefore not only a cardiometabolic priority but a strategic necessity for the whole European clinical research ecosystem.

### Limitations

The present analysis identifies patterns in trial activity but does not directly assess underlying determinants such as regulatory timelines, costs, or infrastructure capacity. Therefore, the interpretations presented in this manuscript should be considered hypothesis-generating.

This study has several strengths, including a comprehensive and transparent methodology, a multi-country comparison, and a disease-specific focus informed by ICD-11 classification. However, limitations include reliance on ClinicalTrials.gov as the sole data source, which may underrepresent non-industry or academic trials not registered in the database, as well as the inclusion of only five selected European countries, which, while conferring homogeneity to the analysis, limits the generalizability of the information and fails to account for potential individual-country level data. Additionally, classification of disease categories and sponsor types required some manual review, which may introduce bias. Finally, the lack of outcome, intervention types, or trial design data limits the ability to assess study quality or success rates.

## Conclusion

The observed heterogeneity in Phase I clinical trial activity on CVDs and CVRFs across Europe calls for coordinated policy action. Without structural reform and strategic investments, Europe risks continued erosion of its role in early-phase drug development. However, by embracing regulatory innovation, reducing infrastructural disparities, and incentivizing collaborative research, the region can reclaim its place as a global leader in clinical innovation.

## Data Availability

The original contributions presented in the study are included in the article/Supplementary Material, further inquiries can be directed to the corresponding author.
