# Supplementary material for: Phase I clinical trials for cardiovascular diseases in Europe: analysis of trends, distribution, and regulatory context
Source: Front Pharmacol. 2026 May 12;17:1774988. doi: 10.3389/fphar.2026.1774988 (PMC13201443; doi:10.3389/fphar.2026.1774988)
Supplement: Supplementary file 1 [file Supplementaryfile1.docx]

**Supplementary material**

**Phase I clinical trials for cardiovascular diseases in Europe: analysis of trends, distribution, and regulatory context**

**ClinicalTrials.gov search strategy**

The registry ClinicalTrials.gov was searched in December 2024 using the Advanced Search interface. Searches were conducted separately for each country and condition/keyword, and the resulting records were exported as CSV datasets.

The following countries were investigated:

- France
- Germany
- Italy
- Spain
- United Kingdom

The following conditions/keywords were used in the search fields Condition or disease and/or Other terms:

- cardiology
- heart disease
- diabetes
- obesity
- endocrinology
- metabolic disorder
- hypercholesterolemia
- hypertension
- hyperglycemia
- hypertriglyceridemia
- dyslipidemia

Each keyword was searched individually with the following filters applied:

- Study type: Interventional studies
- Phase: Early Phase 1 or Phase 1
- Location: one country per query

For each country–keyword combination, two datasets were retrieved, with and without the Healthy Volunteers filter activated.

All retrieved datasets were merged into a single database, resulting in 3,107 records, which were subsequently curated as described in the Methods section of the manuscript.

The study selection process is shown in Supplementary Figure 1.


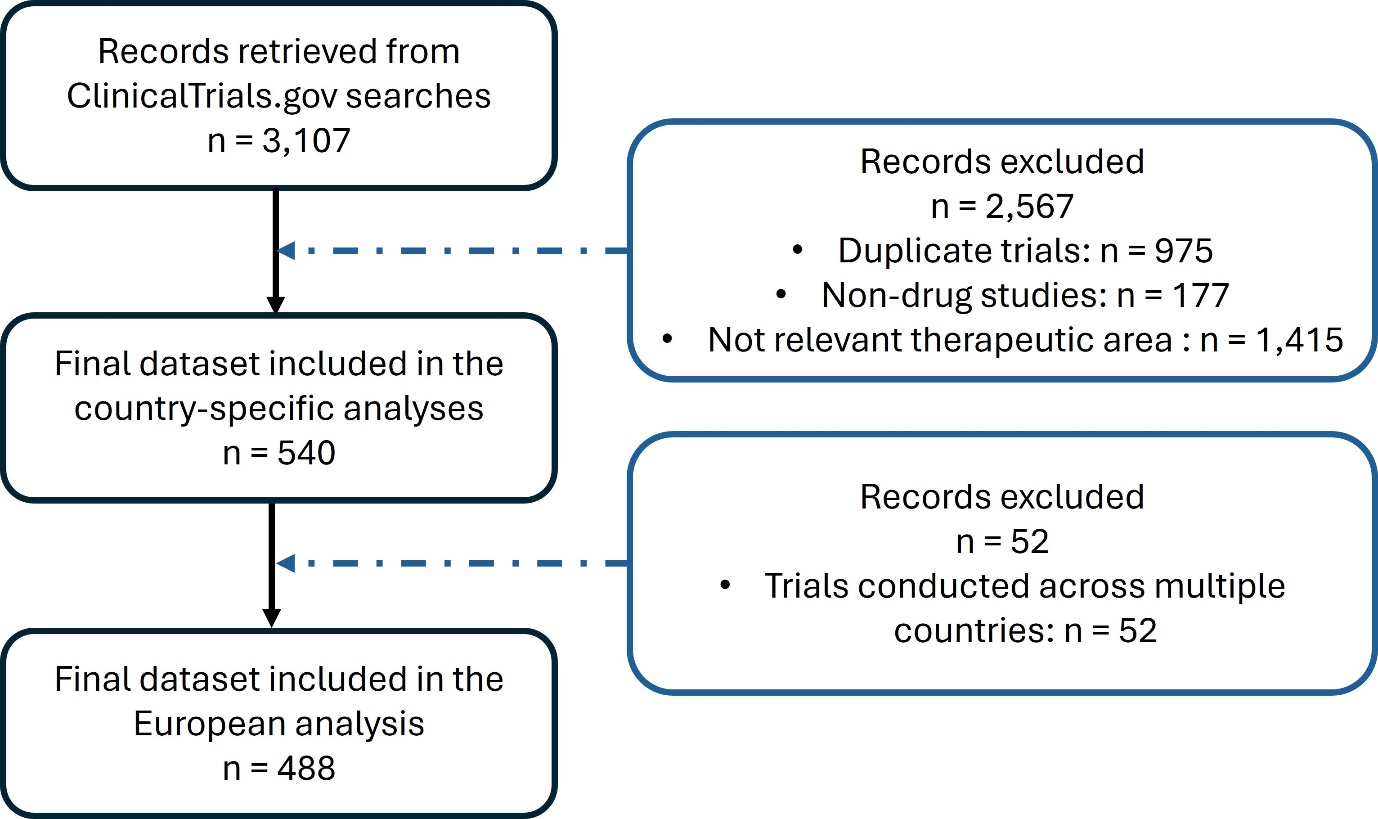


*Supplementary Figure 1 Flow chart of the study selection process*.

| **ICD-11 Chapter and Category** | **ICD-11 Code(s)** | **Abbreviation** | **Included Conditions** |
| --- | --- | --- | --- |
| ***05 Endocrine, nutritional or metabolic diseases*** | | | |
| Disorders of lipoprotein metabolism or certain specified lipidaemias | 5C80 | LIPO | Dyslipidemia |
| Nutritional disorders – Overweight, obesity or specific nutrient excesses | 5B81 | OB | Obesity |
| Endocrine diseases – Diabetes mellitus | 5A11 | D | Diabetes |
| ***11 Diseases of the circulatory system*** | | | |
| Hypertensive diseases | BA00–BA0Z | HYPER | Hypertension |
| Heart failure | BD10 | HF | Heart failure; Hypertension with chronic heart failure; Hypertension with congestive heart failure; Acute coronary syndromes with heart failure |
| Diseases of arteries or arterioles | BD70–BD7Z | DA | Peripheral arterial disease; Critical limb ischemia; Diabetic foot ulcer |
| Diseases of veins | BD80–BD8Z | DV | Thrombotic disease; Chronic venous insufficiency; Venous ulcer |
| Cardiac arrhythmias | BC81–BC8Z | CA | Heart arrhythmias |
| Ischaemic heart diseases | BA40–BA4Z | IHS | Coronary artery disease; Myocardial infarction; Acute myocardial infarction; Acute coronary syndromes |
| Pulmonary heart disease or diseases of pulmonary circulation | BD20–BD2Z | PD | Pulmonary arterial hypertension |
| ***08 Diseases of the nervous system*** | | | |
| Cerebrovascular diseases | 8B00–8B0Z | NEURO | Stroke; Intracerebral haemorrhage |
| ***Other / Multisystem conditions*** | — | Other | Heart transplantation; Cardiovascular disease not otherwise specified |

Supplementary Table 1 Disease classification according to ICD-11 and corresponding abbreviation used in the analysis.
Note: HF, DA, DV, CA, IHS, PD, NEURO and Other are grouped in the macro-category of cardiovascular diseases, whereas LIPO, OB, D and HYPER are included in the macro-category of cardiovascular risk factors.


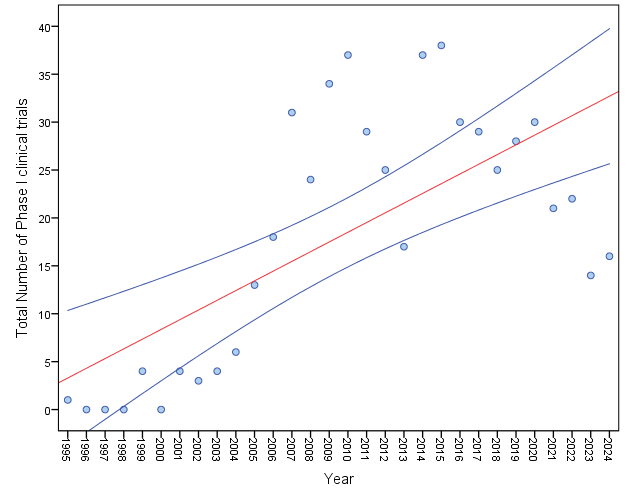


Supplementary Figure 2 Longitudinal linear regression analysis of trend of total number of phase I clinical trials between 1995 and 2024.

| Model Summary | | | | |
| --- | --- | --- | --- | --- |
| Model | R | R Square | Adjusted R Square | Std. Error of the Estimate |
| linear | .685 | .469 | .450 | 9.660 |

| ANOVA | | | | | |
| --- | --- | --- | --- | --- | --- |
| Model | Sum of Squares | df | Mean Square | F | Sig. |
| Regression | 2310.941 | 1 | 2310.941 | 24.763 | < 0.001 |
| Residual | 2613.059 | 28 | 93.324 | - | - |
| Total | 4924.000 | 29 | - | - | - |

|  | Coefficients | | | | | | |
| --- | --- | --- | --- | --- | --- | --- | --- |
| Model | Unstandardized Coefficients | | Standardized Coefficients | t | Sig. | 95.0% CI for B | |
|  | B | Std. Error | Beta |  |  | Lower | Upper |
| (Constant) | -2019.664 | 409.485 | - | -4.932 | < 0.001 | - | - |
| Year | 1.014 | .204 | .685 | 4.976 | < 0.001 | .597 | 1.431 |

Supplementary Table 2 The table reports regression coefficients (β), 95% confidence intervals (CI), coefficient of determination (R²), and p values assessing the association between calendar year and the annual number of registered Phase I clinical trials (1995-2024).

| Model Summary | | | | |
| --- | --- | --- | --- | --- |
| Model | R | R Square | Adjusted R Square | Std. Error of the Estimate |
| Linear | .890 | .792 | .781 | 6.770 |

| ANOVA | | | | | |
| --- | --- | --- | --- | --- | --- |
| Model | Sum of Squares | df | Mean Square | F | Sig. |
| Regression | 3316.369 | 1 | 3316.369 | 72.354 | < 0.001 |
| Residual | 870.869 | 19 | 45.835 | - | - |
| Total | 4187.238 | 20 | - | - | - |

| Coefficients | | | | | | | |
| --- | --- | --- | --- | --- | --- | --- | --- |
| Model | Unstandardized Coefficients | | Standardized Coefficients | t | Sig. | 95.0% CI for B | |
|  | B | Std. Error | Beta |  |  | Lower | Upper |
| (Constant) | -4145.550 | 489.182 | - | -8.474 | < 0.001 | -4145.550 | 489.182 |
| **Year** | 2.075 | .244 | .890 | 8.506 | < 0.001 | 2.075 | .244 |

*Supplementary Table 3 The table reports regression coefficients (β), 95% confidence intervals (CI), coefficient of determination (R²), and p values assessing the trend of total number of Phase I clinical trials between 1995 and 2015*

| Model Summary | | | | |
| --- | --- | --- | --- | --- |
| Model | R | R Square | Adjusted R Square | Std. Error of the Estimate |
| linear | .901 | .812 | .789 | 3.321 |

| ANOVA | | | | | | |
| --- | --- | --- | --- | --- | --- | --- |
| Model | | Sum of Squares | df | Mean Square | F | Sig. |
|  | Regression | 381.894 | 1 | 381.894 | 34.637 | < 0.001 |
|  | Residual | 88.206 | 8 | 11.026 | - | - |
|  | Total | 470.100 | 9 | - | - | - |

| Coefficients | | | | | | | |
| --- | --- | --- | --- | --- | --- | --- | --- |
| Model | Unstandardized Coefficients | | Standardized Coefficients | t | Sig. | 95.0% CI for B | |
|  | B | Std. Error | Beta |  |  | Lower | Upper |
| (Constant) | 4370.285 | 738.281 | - | 5.920 | < 0.001 | - | - |
| Year | -2.152 | .366 | -.901 | -5.885 | < 0.001 | -2.995 | -1.308 |

Supplementary Table 4 The table reports regression coefficients (β), 95% confidence intervals (CI), coefficient of determination (R²), and p values assessing the association between calendar year and the annual number of registered Phase I clinical trials (2015-2024).


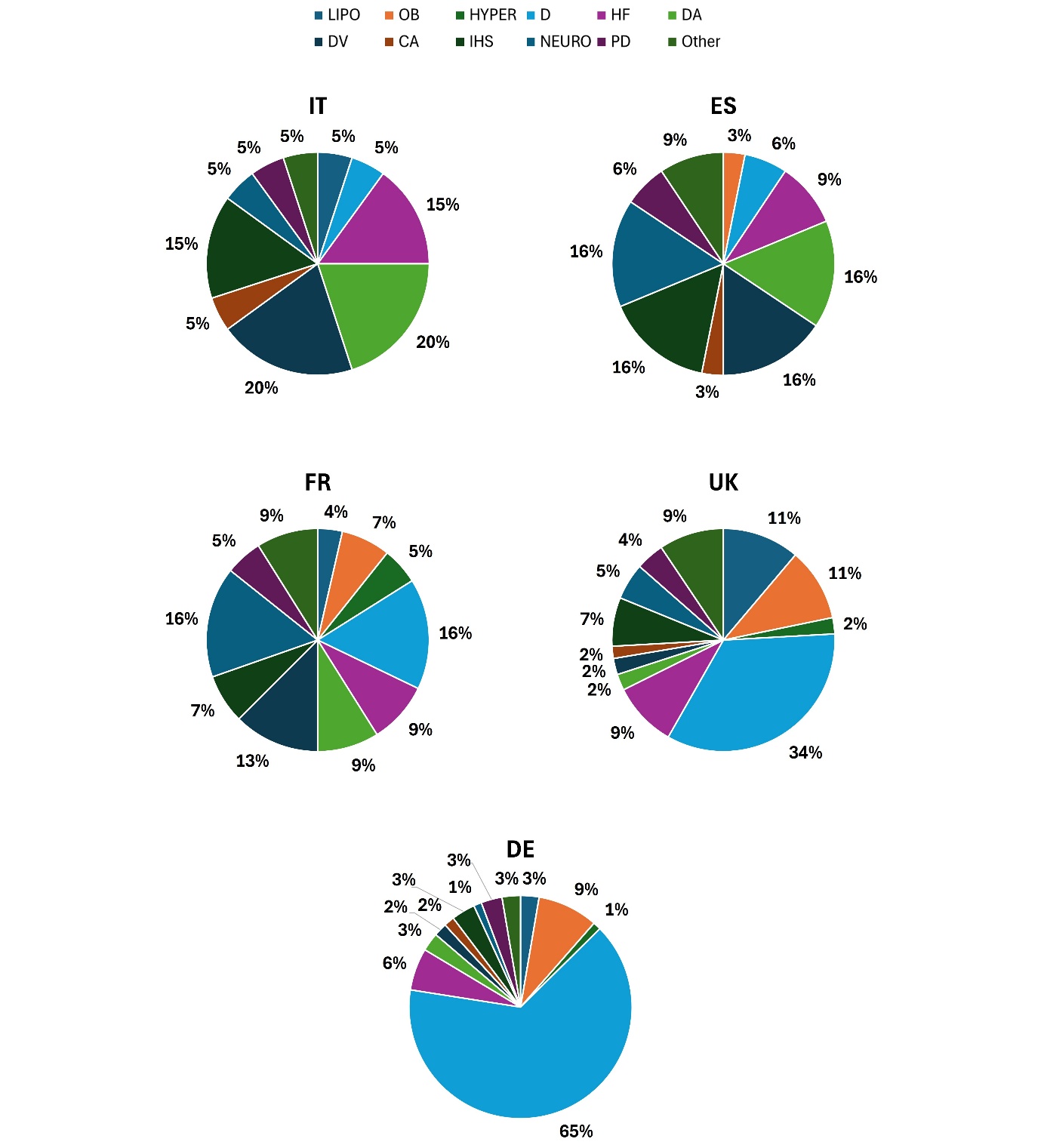


Supplementary Figure 3 Distribution of ICD-11-coded diseases investigated in phase I trials by country.
Abbreviations: LIPO = Disorders of lipoprotein metabolism or certain specified lipidaemias; OB = Nutritional disorders – Overweight, obesity or specific nutrient excesses; D = Endocrine diseases – Diabetes mellitus; HYPER = Hypertensive diseases; HF = Heart failure; DA = Diseases of arteries or arterioles; DV = Diseases of veins; CA = Cardiac arrhythmias; IHS = Ischaemic heart diseases; PD = Pulmonary heart disease or diseases of pulmonary circulation; NEURO = Cerebrovascular diseases.
